# Clustered Multi-Task Learning for Automatic Radar Target Recognition

**DOI:** 10.3390/s17102218

**Published:** 2017-09-27

**Authors:** Cong Li, Weimin Bao, Luping Xu, Hua Zhang

**Affiliations:** School of Aerospace Science and Technology, Xidian University, Xi’an 710126, China; lcongxd@126.com (C.L.); baoweimin@cashq.ac.cn (W.B.); zhanghua@mail.xidian.edu.cn (H.Z.)

**Keywords:** clustered multi-task learning, high-resolution range profile (HRRP), synthetic aperture radar (SAR), radar automatic target recognition (RATR)

## Abstract

Model training is a key technique for radar target recognition. Traditional model training algorithms in the framework of single task leaning ignore the relationships among multiple tasks, which degrades the recognition performance. In this paper, we propose a clustered multi-task learning, which can reveal and share the multi-task relationships for radar target recognition. To further make full use of these relationships, the latent multi-task relationships in the projection space are taken into consideration. Specifically, a constraint term in the projection space is proposed, the main idea of which is that multiple tasks within a close cluster should be close to each other in the projection space. In the proposed method, the cluster structures and multi-task relationships can be autonomously learned and utilized in both of the original and projected space. In view of the nonlinear characteristics of radar targets, the proposed method is extended to a non-linear kernel version and the corresponding non-linear multi-task solving method is proposed. Comprehensive experimental studies on simulated high-resolution range profile dataset and MSTAR SAR public database verify the superiority of the proposed method to some related algorithms.

## 1. Introduction

Automatic target recognition (ATR) systems are used to identify one or a group of target objects in a scene. These ATR systems are to detect and classify targets using various images and signal processing techniques. Due to the capability of producing all-weather, 24-h a day and robustness towards the environmental condition of radar sensors, researchers have drawn much attention on the automatic target recognition based on radar images. Usually, radar images can be divided into one-dimensional high-resolution range profile (HRRP) images and two-dimensional images, like synthetic aperture radar (SAR) images. In recent years, radar images have been intensively studied for ATR in civilian and military fields [1,2,3,4,5,6,7]. Nevertheless, HRRPs and SAR images are sensitive to the variation in the pose and the speckle noise. How to recognize the specified radar targets still requires further study and exploration.

Radar target recognition generally consists of three main separate stages: detection, discrimination, and classification. The first stage aims to approximately determine the location of targets by using the amplitude information of radar signals. The second stage excludes the interference of clutter in background. The last stage is to predict the category of targets using classifiers. In this paper, the classifier design is emphasized. Lots of classical classifiers have been implemented for radar target classification, including k-nearest neighbors (KNN), support vector machine (SVM) [8], AdaBoost [9] and so on. Zhou incorporated the reconstructive power and discriminative power of dictionary atoms for radar target recognition [2]. A complementary spatial pyramid coding (CSPC) approach for radar target recognition was realized by Wang et al. [4]. Song et al. came up with a supervised discriminative dictionary learning (SDDL) method by learning a discriminative dictionary for SAR ATR [6].

Although much work has been done on radar target recognition, two critical problems still exist. To our knowledge, most of the previous methods have been implemented in the framework of single-task learning. Although many sophisticated classifiers have been designed, they cannot make full use of the latent relatedness among multiple radar categories. Therefore, it is challenging to classify the targets with similar patterns by single-task learning methods. On the other hand, the acquired samples are usually limited or imbalanced, since large-scale and complete dataset collection is expensive. Single-task learning can’t realize information (common structures or parameters) sharing, which makes it difficult to train a model. On the contrary, multi-task learning can make use of multi-task relationships and thus help to improve the recognition accuracy. Dong et al. considered three components of monogenic signals as different learning tasks and proposed a joint sparse representation model to exploit the intercorrelation among multiple tasks for SAR ATR [10]. The contrast experiments have proven the superiority of multi-task learning.

A few studies have focused on the multi-task learning for RATR except for [10], even though multi-task learning is superior to single task leaning. Inspired by the recent successful application of multi-task learning in the field of ATR, such as facial expression recognition [11], HEp-2 cell classification [12], and Alzheimer's disease (AD) diagnosis classification [13], we propose a new classification method based on clustered multi-task learning theory. Two issues should be taken into consideration when the clustered multi-task learning method is used for radar target recognition. Due to the target-aspect and pose sensitivity of radar images, the scattering characteristics among different targets in the same target-aspect may be extremely similar, which makes it difficult for humans to cluster radar targets simply using visual appearance. The other problem is that the geometric structure information hidden in the radar images, such as target size and scatterer distribution is complicated and nonlinear, which results in a difficult extraction of cluster information. The multi-task relationship learning (MTRL) method proposed in [14] can autonomously learn the positive and negative task correlations, and also can be easily extended to nonlinear domains, which can address these two issues well. To further improve the classification ability of MTRL, a projection regularization term is added into the objective function to fully explore the cluster structure and multi-task relationships in the original and projected space of model parameter. The projection term assumes that the multiple tasks within a close cluster should be close to each other in the projection space. As an extension of the MTRL method, the proposed method can autonomously learn multi-task relationships, cluster the information of different tasks and be easily extended to nonlinear domains. The main difference between the proposed method and the approach in [10] is that the former can automatically learn the multi-task relationships and clusters hidden in the kernel function space. In terms of object function optimization, SMO method [15] is frequently adopted in the non-linear versions of single-task learning. However, when SMO is used for solving the non-linear extension of multi-task learning, multiple variables need to be processed simultaneously, which greatly increases the heuristic selection time and sometimes can’t guarantee convergence. To overcome this problem, the objective function is firstly transformed into a dual form, and then the widely applied APG method [16] is adopted to solve it. The proposed solving method can guarantee the convergence and be implemented in parallel computing. In addition, extensive studies on the simulated and real databases are conducted to assess and analyze the performance of the proposed method. The proposed method includes the following advantages:

(1) The theory of clustered multi-task learning is applied to radar target recognition.
(2) The potentially useful multi-task relationships in the projection space are taken into consideration, which helps to discriminate the radar targets with similar patterns.
(3) The proposed method can autonomously learn the multi-task relationships, cluster information and be easily extended to nonlinear domains.
(4) APG method is used for solving the non-linear extension of multi-task learning, which guarantees the convergence and can be implemented in parallel computing.

The rest of the paper is organized as follows: in Section 2, the clustered multi-task learning for radar target recognition is proposed. The experimental results and analysis are provided in Section 3, and the paper is finalized with conclusions in Section 4.

## 2. Clustered Multi-Task Learning

### 2.1. Preliminaries

For radar target classification, the model learning for m target categories can be considered as m tasks. During the training phase, a set of training samples ${\left\{\left(x_{j}^{i},y_{j}^{i}\right)\right\}}_{j=1}^{n_{i}}$ are given for model learning, where $x_{j}^{i}\in R^{d}$ is the d-dimension training data, $y_{j}^{i}\in \{0,1\}$ is the label and $n_{i}$ is the number of samples in the ith task. In this paper, the goal is to learn a nonlinear predictive function$f_{i}(x_{j}^{i})=\phi {\left(x_{j}^{i}\right)}^{T}w_{i}+b_{i}$, where $\phi (x_{j}^{i})$ is the nonlinear mapping of sample $x_{j}^{i}$, $w_{i}$ is the model parameter and $b_{i}$ is the offset of ith task. Let $W=[w_{1}\ldots \;w_{m}]$, then the objective function can be formulated as:

(1) $$\underset{W}{\min}f(W)+\Omega (W)$$

where f(W) is the empirical loss function. In this paper, it is defined as:

(2) $$f(W)=\sum_{i=1}^{m}\frac{1}{n_{i}}\sum_{j=1}^{n_{i}}{\left(y_{j}^{i}-\phi {\left(x_{j}^{i}\right)}^{T}w_{i}-b_{i}\right)}^{2}$$

where $n_{i}$ is to alleviate the data imbalance among different tasks. Ω(W) is clustering-based regularization to constrain the shared information among different tasks, and has been the focus of many researchers.

For example, the authors assume that the parameter vector of each task is similar to the average parameter vector, and Ω(W) is formulated as [17]:

(3) $$\Omega (W)=\sum_{i=1}^{m}\sum_{k=1}^{m}\frac{1}{2m}\|{w_{i}-w_{k}\|}_{2}^{2}=tr(WLW^{T})$$

where L is the Laplacian matrix defined on the graph with edge weights equaling to 12m. In [18], the authors assume that all tasks can be grouped into r<m clusters, and Ω(W) is defined as:

(4) $$\Omega (W)=\sum_{c=1}^{r}\sum_{v\in I_{c}}^{}\|{w_{v}-{\bar{w}}_{c}\|}_{2}^{2}=tr(W^{T}W)-tr(F^{T}W^{T}WF)$$

where $I_{c}$ is the index set of the cth cluster, ${\bar{w}}_{j}$ denotes the mean of the cth cluster, and matrix $F\in R^{m\times r}$ is an orthogonal cluster indicator matrix with $F_{i,c}=1/\sqrt{n_{c}}$ if $i\in I_{c}$ and $F_{i,c}=0$ otherwise. These methods assume that the cluster structures or the multi-task relationships are known. Nevertheless, sometimes these model assumptions may be incorrect or even worse. Thus, learning the task relationships from data automatically is a more favorable choice. In [14], a multi-task relationship learning (MTRL) method is proposed, which can autonomously learn the positive and negative task correlation. The Ω(W) is given as:

(5) $$\Omega (W)=tr(WS^{-1}W^{T})\;s.t.\;S\underline{≻}0,\;tr(S)=1$$

where  S is defined as a task covariance matrix and W is the model parameter.

### 2.2. Proposed Clustered Multi-Task Learning

In MTRL method, the multi-task relationships among different mode parameters are fully utilized. To further improve the classification performance of MTRL, we assume that the tasks with a close relationship should be close to each other in the projection space $X^{T}W$. That is to say, the task covariance matrix S should reflect the multi-task relationships in the original and projected space of mode parameters. The proposed Ω(W) can be formulated as:

(6) $$\begin{array}{l} \Omega (W)=\frac{\lambda_{1}}{2}\|{W\|}_{2}^{2}+\frac{\lambda_{2}}{2}tr(WS^{-1}W^{T})\;+\frac{\lambda_{3}\;}{2}tr(PS^{-1}P^{T})\;\\ \;s.t.\;S\underline{≻}0,\;tr(S)=1\;,\;p_{j}^{i}={\left(x_{j}^{i}\right)}^{T}w_{i} \end{array}$$

where $P=[P_{1},\ldots ,P_{m}]$, $P_{i}=[p_{1}^{i},\ldots ,p_{n_{i}}^{i}]$, $\lambda_{i}$ is the regularization parameter, and S denotes the task covariance matrix. The target features hidden in the radar images are usually nonlinear. Thus a nonlinear kernel version of the proposed method is obtained:

(7) $$\Omega (W)\;=\frac{\lambda_{1}}{2}\|{W\|}_{2}^{2}+\frac{\lambda_{2}}{2}tr(WS^{-1}W^{T})+\frac{\lambda_{3}}{2}tr(PS^{-1}P^{T})$$

where $P=[P_{1},\ldots ,P_{m}]$, $P_{i}=\phi {\left(X_{i}\right)}^{T}w_{i}$, and $\phi (X_{i})=[\phi (x_{1}^{i}),\ldots \;,\phi (x_{n_{i}}^{i})]$. The first term penalizes the complexity of W. The second term restricts the distance between $w_{i}$ and $w_{k}$ in the model parameter space, and the third term controls the distance between $\phi (X_{i})$ and $\phi (X_{k})$ in the projected space. The latter two regularization terms imply that the distance between a pair of task $T_{i}$ and $T_{k}$ should be as small as possible in the original and projected space if they belongs to the same cluster. To sum up, the objective function can be denoted as:

(8) $$\begin{array}{l} \underset{W,b}{\min}\sum_{i=1}^{m}\frac{1}{n_{i}}\sum_{j=1}^{n_{i}}{\left(\xi_{j}^{i}\right)}^{2}+\frac{\lambda_{1}}{2}\|{W\|}_{2}^{2}+\frac{\lambda_{2}}{2}tr(WS^{-1}W^{T})+\frac{\lambda_{3}}{2}tr(PS^{-1}P^{T})\\ \;s.t.\;\xi_{j}^{i}=y_{j}^{i}-\phi {\left(x_{j}^{i}\right)}^{T}w_{i}-b_{i},\;p_{j}^{i}=\phi {\left(x_{j}^{i}\right)}^{T}w_{i} \end{array}$$

### 2.3. Proposed Optimization Method

The objective function of problem (8) is convex on all variables. But it is not easy to optimize the objective function with respect to all the variables simultaneously. Here an alternating method is adopted to solve the problem. Firstly, W and b are updated with fixed S. Then S is updated with fixed W and b. 

Specifically, when S is fixed, the optimization problem for updating W and b can be stated as:

(9) $$\begin{array}{l} \underset{W,b}{\min}\sum_{i=1}^{m}\frac{1}{n_{i}}\sum_{j=1}^{n_{i}}{\left(\xi_{j}^{i}\right)}^{2}+\frac{\lambda_{1}}{2}\|{W\|}_{2}^{2}+\frac{\lambda_{2}}{2}tr(WS^{-1}W^{T})+\frac{\lambda_{3}}{2}tr(PS^{-1}P^{T})\\ \;s.t.\;\xi_{j}^{i}=y_{j}^{i}-\phi {\left(x_{j}^{i}\right)}^{T}w_{i}-b_{i},\;p_{j}^{i}=\phi {\left(x_{j}^{i}\right)}^{T}w_{i} \end{array}$$

To facilitate a kernel extension for proposed method, the optimization problem is transformed into a dual form:

(10) $$\begin{array}{l} L=\sum_{i=1}^{m}\frac{1}{n_{i}}\sum_{j=1}^{n_{i}}{\left(\xi_{j}^{i}\right)}^{2}+\frac{\lambda_{1}}{2}\|{W\|}_{2}^{2}+\frac{\lambda_{2}}{2}tr(WS^{-1}W^{T})+\frac{\lambda_{3}}{2}tr(PS^{-1}P^{T})\\ \;+\sum_{i=1}^{m}\sum_{j=1}^{n_{i}}\alpha_{j}^{i}{\left(y_{j}^{i}-\phi {\left(x_{j}^{i}\right)}^{T}w_{i}-b_{i}-\xi_{j}^{i}\right)}^{2}+\sum_{i=1}^{m}\sum_{j=1}^{n_{i}}\beta_{j}^{i}{\left(p_{j}^{i}-\phi {\left(x_{j}^{i}\right)}^{T}w_{i}\right)}^{2} \end{array}$$

where $\alpha_{j}^{i}$ and $\beta_{j}^{i}$ are the Lagrange multipliers associated with the jth training sample of the ith task. Setting the derivative of L with respect to W, $b_{i}$, $\xi_{j}^{i}$ and P equal to zero, we obtain:

(11) $$\begin{array}{l} \frac{\partial L}{\partial W}=\lambda_{1}\cdot W+\lambda_{2}\cdot WS^{-1}-\sum_{i=1}^{m}\sum_{j=1}^{n_{i}}\alpha_{j}^{i}\phi (x_{j}^{i})e_{i}{}^{T}-\sum_{i=1}^{m}\sum_{j=1}^{n_{i}}\beta_{j}^{i}\phi (x_{j}^{i})e_{i}{}^{T}=0\\ \frac{\partial L}{\partial b_{i}}=-\sum_{j=1}^{n_{i}}\alpha_{j}^{i}=0\\ \frac{\partial L}{\partial \xi_{j}^{i}}=\frac{2}{n_{i}}\xi_{j}^{i}-\alpha_{j}^{i}=0\\ \frac{\partial L}{\partial P}=\lambda_{1}\cdot PS^{-1}+B=0 \end{array}$$

where $e_{i}$ is the ith column vector of $I_{m\times m}$ and $\beta_{j}^{i}$ is the (j,i)th element of B. Plugging Equation (11) into Equation (10), the following form is obtained

(12) $$E=\underset{\alpha ,\beta }{\max}\sum_{i=1}^{m}\sum_{j=1}^{n_{i}}\alpha_{j}^{i}y_{j}^{i}-\frac{n_{i}}{4}\sum_{i=1}^{m}\sum_{j=1}^{n_{i}}{\left(\alpha_{j}^{i}\right)}^{2}-\frac{1}{2}\alpha^{T}K\alpha -\frac{1}{2}\alpha^{T}K\beta -\frac{1}{2}\beta^{T}K\alpha -\frac{1}{2}\beta^{T}K\beta -\frac{1}{2\lambda_{3}}\beta^{T}\tilde{S}\beta$$

where $\alpha \;=\;(\alpha_{1}^{1},\ldots ,\alpha_{n_{}}^{m}{)}^{T}$ and $\beta ={\left(\beta_{1}^{1},\ldots ,\beta_{n_{}}^{m}\right)}^{T}$. $K\in R^{\sum_{i=1}^{m}n_{i}\times \sum_{i=1}^{m}n_{i}}$ is the multi-task kernel matrix defined on all the training samples. For any two training samples $\left(x_{j_{1}}^{i_{1}},x_{j_{2}}^{i_{2}}\right)$, the corresponding multi-task kernel is referred to as $K(x_{j_{1}}^{i_{1}},x_{j_{2}}^{i_{2}})=e_{i_{1}}^{T}{\left(\lambda_{1}I_{m\times m}+\lambda_{2}L\right)}^{-1}e_{i_{2}}\phi {\left(x_{j_{1}}^{i_{1}}\right)}^{T}\phi (x_{j_{2}}^{i_{2}})$. $\tilde{S}\in R^{\sum_{i=1}^{m}n_{i}\;\times \;\sum_{i=1}^{m}n_{i}}$ contains m × m sub-matrices and the (i,j)th sub-matrix is a diagonal matrix with diagonal element S(i,j). Since problem (12) is an unconstrained optimization problem with respect to β, the derivative of E with respect to β can be given as:

(13) $$\frac{\partial E}{\partial \beta }=-K\alpha -K\beta -\frac{\tilde{L}}{\lambda_{3}}\beta$$

Setting the derivative equal to zero, we obtain:

(14) $$\beta =Q^{-1}K\alpha$$

where $Q=-(K+\frac{\tilde{S}}{\lambda_{3}})$. Substituting Equation (14) into Equation (12), we can obtain:

(15) $$\underset{\alpha }{\min}\frac{1}{2}\alpha^{T}\tilde{K}\alpha -\sum_{i=1}^{m}\sum_{j=1}^{n_{i}}\alpha_{j}^{i}y_{j}^{i}$$

where $\tilde{K}=K+2KQ^{-1}K+KQ^{-1}KQ^{-1}K+1/\lambda_{3}KQ^{-1}\tilde{L}Q^{-1}K+1/2\Lambda$, and $\Lambda \in R^{\sum_{i=1}^{m}n_{i}\;\times \;\sum_{i=1}^{m}n_{i}}$ is a diagonal matrix with diagonal element $n_{i}$ if the corresponding data point is from the ith task. 

So far, the problem (9) is converted to a familiar form. In most literatures [14,19], Equation (15) is solved by an SMO algorithm, being similar to the least-squares SVM [15]. However, multiple variables need to be heuristically selected, when SMO method is used for solving the non-linear extension of multi-task learning. In this paper, the SMO approach can’t guarantee convergence due to the mutual interference when multiple variables are heuristically selected. In the proposed solving method, the problem (15) is efficiently solved by the widely applied APG method [16]. Specifically, the objective function is divided into the smooth part $f(\alpha )=\frac{1}{2}\alpha^{T}\tilde{K}\alpha$ and non-smooth part $g(\alpha )=-\sum_{i=1}^{m}\sum_{j=1}^{n_{i}}\alpha_{j}^{i}y_{j}^{i}$. The gradient of f(α) is Lipschitz continuous with the Lipschitz constant ℓ satisfying with $\ell \geq {\|\tilde{K}\|}_{2}$. Given ℓ, a surrogate function $T_{\ell }(\alpha ,\alpha_{k})$ is defined as:

(16) $$T_{\ell }(\alpha ,\alpha_{k})=f(\alpha_{k})+<\alpha -\alpha_{k},\nabla f(\alpha_{k})>+\frac{\ell }{2}{\|\alpha -\alpha_{k}\|}_{2}^{2}+g(\alpha )$$

where $\nabla f(\alpha_{k})$ denotes the derivative of f(α) with respect to α at $\alpha =\alpha_{k}$. After we omitted the constant terms, Equation (16) can be redefined as:

(17) $$M_{\ell }(\alpha_{k})=\underset{\alpha }{\min}\frac{\ell }{2}{\|\alpha -\alpha_{k}\|}_{2}^{2}+\alpha^{T}\tilde{K}\alpha_{k}-\sum_{i=1}^{m}\sum_{j=1}^{n_{i}}\alpha_{j}^{i}y_{j}^{i}\;s.t.\;\sum_{j=1}^{n_{i}}\alpha_{j}^{i}=0\;\forall i$$

which can be decoupled into m subproblems with the ith one formulated as:

(18) $$M_{\ell }({\left(\alpha_{k}\right)}_{i})=\underset{\alpha }{\min}\frac{\ell }{2}{\|\alpha_{i}-{\left(\alpha_{k}\right)}_{i}\|}_{2}^{2}+\alpha_{i}{}^{T}z_{i}-\sum_{i=1}^{m}\sum_{j=1}^{n_{i}}\alpha_{j}^{i}y_{j}^{i}\;s.t.\;\sum_{j=1}^{n_{i}}\alpha_{j}^{i}=0\;$$

where $z=\tilde{K}\alpha_{k}$ and $z_{i}$ is a subvector of z corresponding to the ithtask. It is not difficult to see that Equation (17) can be easily implemented in parallel computing. Based on the Lagrange multiplier method, we can obtain the analytical solution of problem (17) as follows:

(19) $$\alpha =\frac{1}{\ell }(Y-D)+V$$

where $Y={\left(y_{1}^{1},\ldots ,y_{n_{m}}^{m}\right)}^{T}$, $V={\left(v_{1}^{1},\ldots ,v_{n_{m}}^{m}\right)}^{T}$, vi=(v1i,…,vnii)T=(αk)i−ziℓ, $D={\left(d_{1}^{1},\ldots ,d_{n_{m}}^{m}\right)}^{T}$, and $d_{j}^{i}=\frac{1}{n_{i}}\sum_{j=1}^{n_{i}}\ell \cdot v_{j}^{i}+y_{j}^{i}$.

When W and b are obtained, the subproblem for minimizing Equation (10) over S can be stated as:

(20) $$\begin{array}{l} \underset{S}{\min}\;tr(\lambda_{2}tr(WS^{-1}W^{T})+\lambda_{3}tr(PS^{-1}P^{T}))\;\\ \;s.t.\;S\underline{≻}0,\;tr(S)=1 \end{array}$$

Similar to [14], the analytical solution S is given as:

(21) S=(λ2WTW+λ3PTP)12tr((λ2WTW+λ3PTP)12)

**Figure 1:**
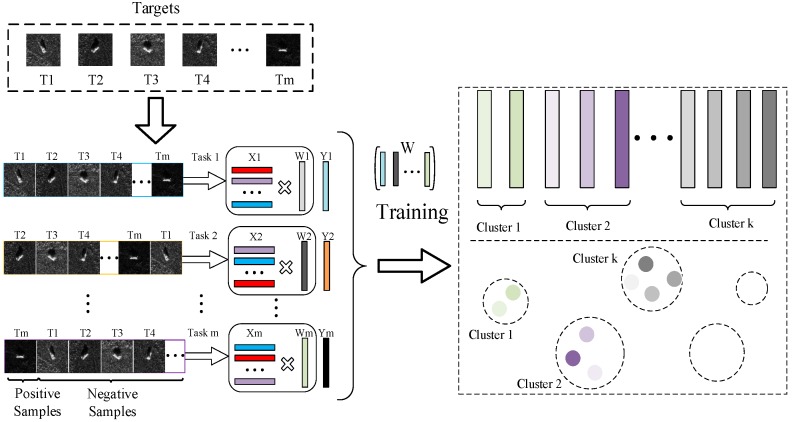
Illustration of proposed method. Firstly, m tasks are formed by concatenating the m targets. In each of the task, the first target is considered as positive sample, and the others negative ones. Then, multi-task learning is performed under the constraint of objective function Equation (8). After training, the multi-task relationships and clustered structures are obtained by Equation (21). In the figure, tasks with similar colors are similar with each other. Finally, the decision is reached by the obtained nonlinear predictive function Equation (22).

Then the nonlinear predictive function $f_{i}(x_{j}^{i})=\phi {\left(x_{j}^{i}\right)}^{T}w_{i}+b_{i}$ can be obtained:

(22) $$f_{i}(x_{j}^{i})=\sum_{i_{0}=1}^{m}\sum_{j_{0}=1}^{n_{i}}(\alpha_{j_{0}}^{i_{0}}+\beta_{j_{0}}^{i_{0}})K(x_{j_{0}}^{i_{0}},x_{j}^{i})+\frac{1}{n_{i}}\sum_{j=1}^{n_{i}}\left(\frac{n_{i}\alpha_{j}^{i}}{2}+T_{j}^{i}\right)$$

where $T_{j}^{i}=\alpha^{T}{\tilde{k}}_{j}^{i}-y_{j}^{i}$ and ${\tilde{k}}_{j}^{i}$ is a column of $\tilde{K}$ corresponding to $x_{j}^{i}$. The proposed method can be pictorially shown in Figure 1, and the corresponding recognition procedure is given in Algorithm 1.

## 3. Experimental Results and Analysis

In order to verify the effectiveness and robustness of the proposed method, simulated HRRP datasets and MSTAR SAR public databases are used for tests. In the following studies, a one-against-all framework is adopted, where each one-against-all classification problem is considered as a task. To quantitatively assess the performance, several state-of-the-art algorithms, including KNN, SVM and some other multi-task learning methods, which are summarized in Table 1, are used as the reference.

**Algorithm 1.** Pseudo Code for Solving Problem (8)1: Input $X\in R^{d\times \sum_{i=1}^{m}n_{i}}$, $Y\in R^{\sum_{i=1}^{m}n_{i}\times 1}$, $\lambda_{1}$, $\lambda_{2}$, $\lambda_{3}$; 2: Initialize $S=I^{m\times m}$, $W=0^{{}^{d\times m}}$ and $b=0^{{}^{1\times m}}$; 3: while not converged 4:  Update W and b 5:    Reformulate the optimize problem (9) into a dual form (12) 6:    Update β by Equation (14) 7:    Solving problem (15) by using the APG method 8:  Update S by using Equation (21) 9: end while 10: Output W, b and S.

### 3.1. Investigations Based on a Simulated Database

**Figure 2:**
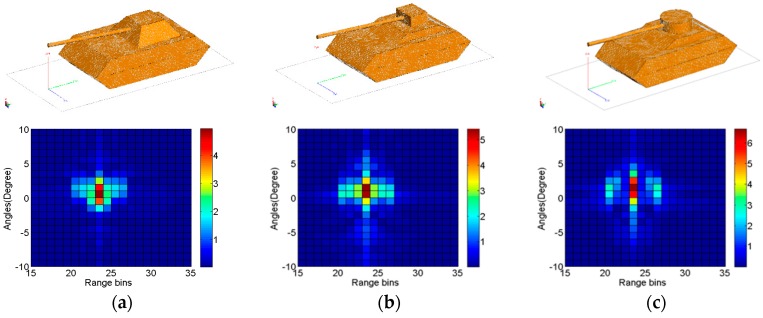
Geometrical models (top) and HRRPs (bottom) of three tanks. (**a**) Tank 1; (**b**) Tank 2; (**c**) Tank 3.

In the simulation experiments, three categories of tank models are considered as the radar targets. HRRP samples are obtained by performing an IFFT of RCS samples, which are generated by FEKO software, whose electromagnetic simulation parameters are listed in Table 2. The three targets and their corresponding HRRPs are shown in Figure 2. In the simulation, the profiles generated at the depression angle of 15° are randomly divided into two equal parts, one for training and the other for testing. For each target, a 50-dimensional feature is achieved by principal component analysis (PCA). In this section, the influences of model parameters on the recognition performance are discussed, then two comparative experiments are conducted. In each of the numerical simulation, twenty times Monte Carlo simulation experiments are performed to achieve the average recognition results.

#### 3.1.1. Influence of Model Parameters

In the prosed model, there are three parameters $\lambda_{1}$, $\lambda_{2}$ and $\lambda_{3}$. Here we set $\lambda_{1}=1$, and only consider the influence of parameters $\lambda_{2}\in \{10^{-1},10^{-2},10^{-3},10^{-4},10^{-5}\}$ and $\lambda_{3}\in \{10^{-2},10^{-3},10^{-4},10^{-5},10^{-6}\}$.

**Figure 3:**
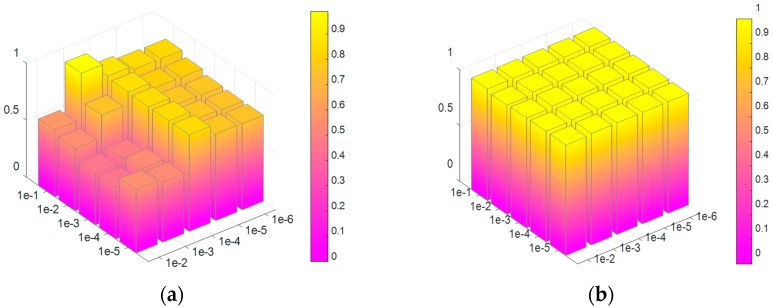
Average results of ACC and AUC versus parameter $\lambda_{2}$ and $\lambda_{3}$. (**a**) ACC results. (**b**) AUC results.

Two metrics of ACC (accuracy) and AUC (area under curve) are adopted to validate the proposed method. The average results are shown in Figure 3. Figure 3a shows that the best recognition rate is reached when $\lambda_{2}$ and $\lambda_{3}$ are equal to $10^{-1}$ and $10^{-3}$, respectively. The optimal value of $\lambda_{3}$ is less than that of $\lambda_{2}$, when our method achieves the best performance. This indicates that the distances among three tasks in the model parameter space are larger than that in the projected space. The penalty of the distance in model parameter space is severer than that in projected space if $\lambda_{2}>\lambda_{3}$, which promotes the generalization ability. Figure 3b shows that the value of AUC approximately equals to one in most of the combinations of $\lambda_{2}$ and $\lambda_{3}$, which means that our model has a strong sorting ability for the samples. Furthermore, an ideal AUC value combined with a worse ACC value denotes that many of the negative samples are classified as positive samples. That’s because the negative and positive samples are unbalanced. For example, in the task of tank 1 against tank 2 and tank 3 classification, the samples of tank 1 are set as positive, and tank 2 and tank 3 are set as negative ones, which leads to a sample unbalanced problem. However, when appropriate combination of $\lambda_{2}$ and $\lambda_{3}$ is chosen, the imbalance problem of samples will disappear.

#### 3.1.2. Comparison of Single Task and Multiple Task

**Figure 4:**
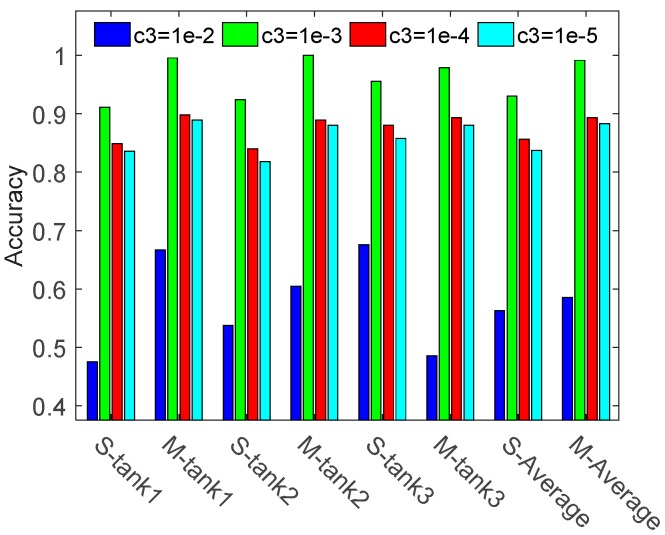
Recognition rate of three tanks when using different parameters $\lambda_{3}\;(c3)$ . The term ‘S-tank1’ (‘M-tank1’) denotes the recognition rate of tank1 in the framework of single (multiple) task learning, and ‘S-Average’ (‘M-Average’) means the average results of three tanks.

To verify the effectiveness of multi-task learning, single task learning and multiple (three) tasks learning methods are compared, where parameter $\lambda_{1}$ is set as 1, $\lambda_{2}$ for $10^{-1}$ and $\lambda_{3}\;(c_{3})$ for $\left\{10^{-2},10^{-3},10^{-4},10^{-5}\right\}$. The ACC results of three tanks are shown in Figure 4.

**Figure 5:**
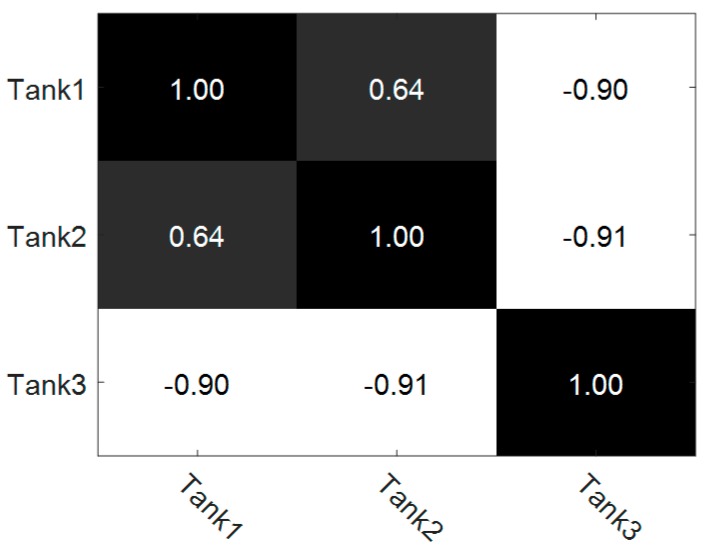
Correlation coefficients of three different tasks. The term ‘Tank1’ (‘Tank2’, ‘Tank 3’) denotes the task to classify tank 1 (tank 2, tank 3).

As shown in Figure 4, the overall recognition rate of three tasks jointly training method is 0.5856, 0.9915, 0.8933 and 0.8830 respectively when using different parameter $\lambda_{3}$. It is 2.26%, 6.11%, 3.70% and 4.59% better than that of the single task learning method. The result denotes that by jointly learning three tasks we can reveal and share the relationships among different tasks, which helps to discriminate the tanks with similar patterns. In our method, the relationships among different tasks can be automatically achieved by computing the learned covariance matrix S. The correlation coefficients of three tasks are shown in Figure 5, when $\lambda_{3}$ equals $10^{-3}$.

The relationship matrix shows that task 1 and task 2 have a high negative correlation with task 3. In the proposed three tasks jointly learning method, these relationships can be accurately described and utilized in the parameter space and projected space. Therefore, a 99.15% average recognition accuracy is achieved by this method. However, when these relationships are not properly handled, these strong correlations will degrade the model learning. Figure 4 shows that when $\lambda_{3}$ equals $10^{-2}$, the ACC of tank 3 by multi-task learning is poor. The reason is that when a tight constraint is imposed on the projected space, the generalization ability of model will decline, which makes it difficult to accurately classify the highly correlated tank 3. Nevertheless, in most of the parameters $\lambda_{3}$, the performance of multi-task learning is better than that of single task learning.

#### 3.1.3. Comparison against the State of the Art

**Figure 6:**
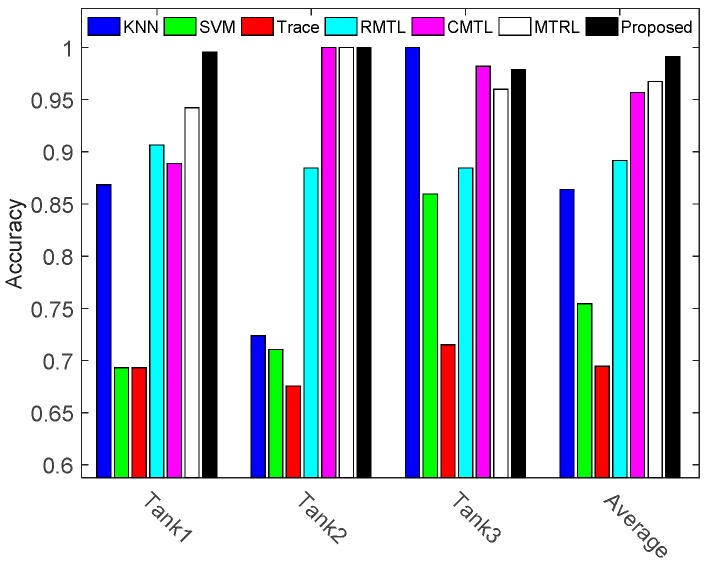
Recognition rates of all the compared methods. Term ‘Average’ denotes the average recognition rate of three tanks.

To evaluate the performance of proposed method, our method is compared with the reference methods and the results are shown in Figure 6 and Table 3.

Figure 6 shows that the multi-task learning with a trace-norm regularization has the lowest recognition rate. It is because that the trace method learns a linear predictive function, which can’t accurately describe the nonlinear structures of HRRP data. This result suggests that it is necessary to extend the multi-task learning methods to nonlinear domains. Table 3 shows that the overall recognition rate for our method is 0.9915, compared to 0.9674 for MTRL, 0.9570 for CMTL, 0.8918 for RMTL, 0.6944 for Trace, 0.7543 for SVM and 0.8640 for KNN. It is 2.41%, 3.45%, 9.97%, 29.71%, 23.72% and 12.75% better than the competitors, MTRL, CMTL, RMTL, Trace, SVM and KNN, respectively. The simulation results denote that our method can accurately describe and utilize the three tasks relationships in the parameter space and projected space, which helps improve the recognition rate of radar targets with highly similar patterns.

### 3.2. Investigations Based on MSTAR Database

To further verify the effectiveness of the proposed method, extensive studies have been done based on the MSTAR public database, a gallery collected using a 10-GHz SAR sensor with 1 ft × 1 ft resolution in range and azimuth. Images are captured at various depressions over 0–359° range of aspect view. The sizes of the images are all around 128×128 pixels. To further avoid the influence of clutter, the images are cropped to 64×64 pixels. In this paper, the intensity of raw image is adopted as the feature. Specially, each raw image is concatenated into a 4096-dimensinal long vector. Then a 200-dimensional feature is achieved by PCA method. In the following numerical simulations, the parameters $\lambda_{1}$, $\lambda_{2}$ and $\lambda_{3}$ of our method are set as 1, 0.1 and 0.001, respectively.

#### 3.2.1. Target Recognition under Standard Operating Conditions (SOC)

**Figure 7:**
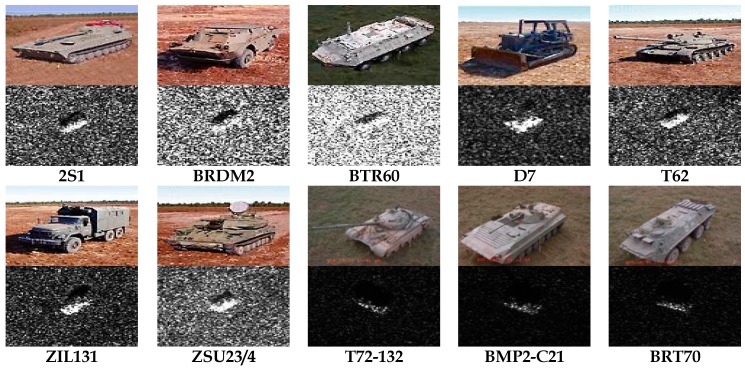
The optical and SAR images of ten targets to be recognized. The descriptions of these vehicles can be referred to [21].

We first consider target recognition under SOC. Images acquired under operating condition of a 17° depression angle are used to train the mode, while the ones captured at an operating condition of a 15° depression angle are used for testing, as shown in Table 4. All ten targets are employed and their optical and SAR images are given in Figure 7. Among these vehicles, BMP2 and T72 have several variants with small structural modifications (denoted by series number). Only the standards (SN_9563 for BMP2 and SN_132 for T72) captured at 17° depression are available for training.

(A) Comparison of Single Task Learning and Multi-task Learning

**Figure 8:**
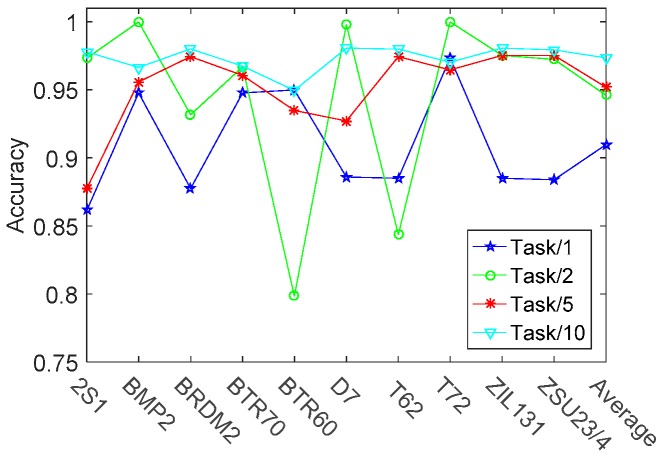
The recognition rates of ten vehicles with different learning methods. The term ‘Task/1’ denotes that each of the ten modes (classifiers) is individually learned (trained). While the term l ‘Task/2’ (‘Task/5’, ‘Task/10’) means that every two (five, ten) modes are jointly learned.

**Figure 9:**
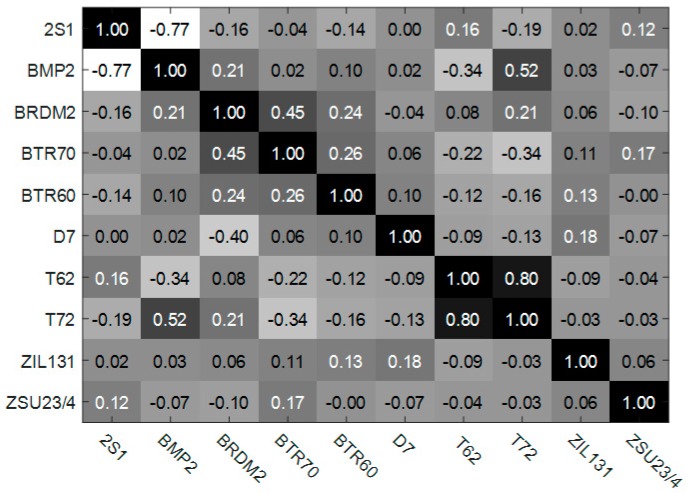
Correlation coefficients of ten different tasks. The term’2S1’means the task to recognize vehicle 2S1 and the meanings of other terms are similar to this one.

In this section, single task learning method is compared with the other training method, like two tasks jointly learning, five tasks jointly learning, and ten tasks jointly learning method. Taking five tasks jointly learning method as an example, it is realized by diving the ten tasks into two equal groups and the tasks within the same groups are jointly learning. The formation of the other multi-task leaning method is similar to this one. The recognition results with different training methods are shown in Figure 8. Besides, the learned ten tasks relationships matrix is shown in Figure 9. As shown in Figure 8, the overall recognition rate of ten targets by ten modes jointly training is increased by 6.37%, 2.74%, and 2.14% respectively, compared with one mode individually training, two modes jointly training and five models jointly training. Moreover, ten modes jointly learning has a more robust ACC result compared with the other training methods. This is due to the fact that ten modes jointly learning method imposes a unified sparse constraint on all of the ten tasks and achieves a global balance in the process of training the ten models. Figure 9 shows that two groups of tasks (‘2S1’ and ‘BMP2’, ‘T62’ and‘T72’) are highly correlated. The single task leaning method can’t appropriately handle and utilize these relationships, thus not being able to get a recognition rate as well as the multi-task leaning method.

(B) Comparison against the State of the Art

**Figure 10:**
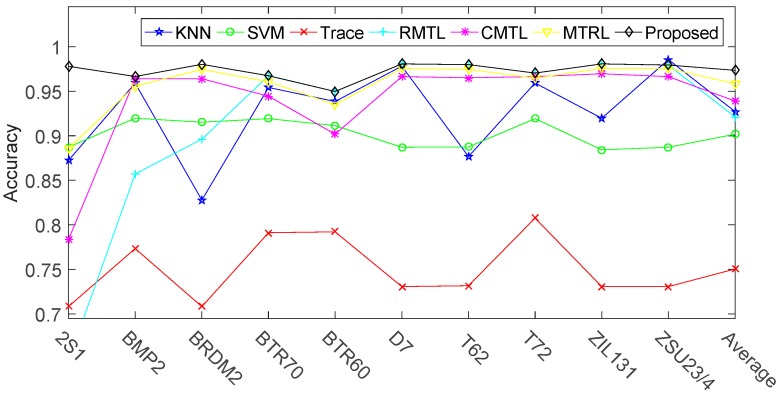
Recogniton rates of ten vehicles versus different methods.

Figure 10 and Table 5 show the comparison ACC results of the proposed method and the reference methods. From Table 5, we can see that the overall recognition rate of our method is 0.9734, compared to 0.9584 for MTRL, 0.9391 for CMTL, 0.9209 for RMTL, 0.7504 for Trace, 0.9017 for SVM and 0.9271 for KNN. It is 1.50%, 3.43%, 5.25%, 22.30%, 7.17% and 4.63% better than the competitors, MTRL, CMTL, RMTL, Trace, SVM and KNN, respectively. The results show that jointly training the ten modes under a unifying classification framework is beneficial for the improvement of recognition rate. Besides, Figure 10 also shows that the ACC of target ‘2S1’ is lower than other targets in most of the reference methods. This may be because that, as shown in Figure 9, tasks ‘2S1’ and ‘BMP2’ are highly correlated and most of the reference methods can’t accurately describe and utilize this relationship, which results in a recognition performance degradation. On the contrary, the proposed method can utilize this relationship and get a better recognition accuracy.

#### 3.2.2. Target Recognition under Extended Operating Conditions (EOC)

To assure the practicability of our method, the recognition performances under different depression angles are assessed. Three vehicle targets 2S1, BRDM2, and ZSU23/4 are utilized. The images captured at an operating condition of a 17° depression are used to train the algorithm, while the ones collected at an operating condition of 30° and 45° depressions are used for testing, as shown in Table 6.

(A) Comparison of Single Task Learning and Multi-task Learning

**Figure 11:**
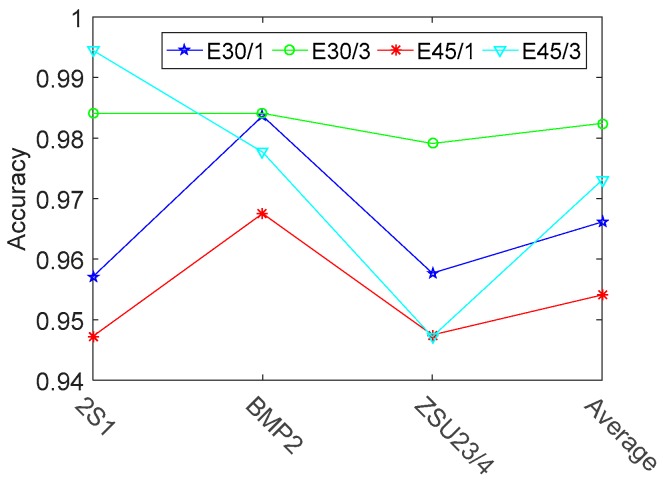
Recognition rates of three vehicles with different learning methods. The term ‘E30/1’ (‘E45/1’) denotes that each of the three modes is individually learned at an operating condition of a 17° depression and tested at an operating condition of 30° (45°) depression. Similar to the terms ‘E30/1’ and ‘E45/1’, the term ‘E30/3’ (‘E45/3’) denotes that all of the three modes are jointly learned.

**Figure 12:**
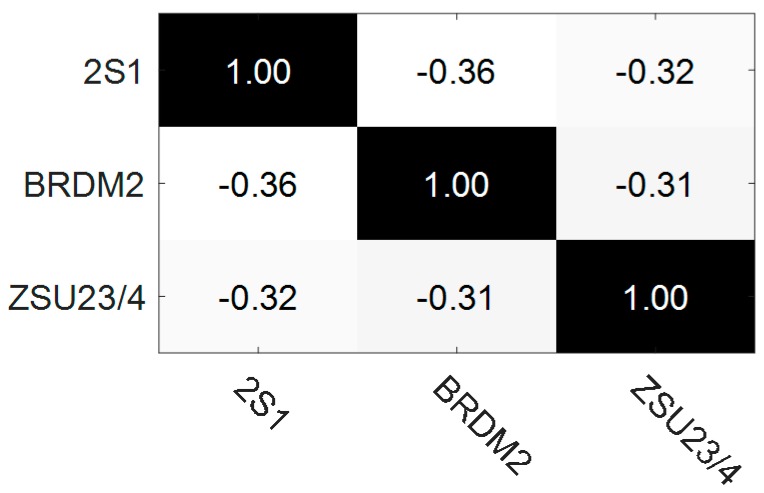
Correlation coefficients of three different tasks. The term’2S1’means the task to recognize vehicle 2S1 and the meanings of other terms are similar to this one.

In this section, single task learning is compared with three tasks jointly learning. The ACC results and correlation coefficients matrix of three vehicles are shown in Figure 11 and Figure 12 respectively.

Figure 11 indicates that the overall recognition rate of three modes jointly learning is 1.63% and 1.90% better than that of single mode individually learning under 30° and 45° testing depression angles, respectively. Figure 12 shows that the three tasks are connected with each other. The tasks connections in EOC test are not close enough, when compared with the relationships shown in Figure 5. One reason is that the SAR images contain a lot of speckle noises, while the simulated HRRP data does not contain noises. Nevertheless, the improvements of overall recognition rate are significant by means of three tasks jointly learning in both of the MSTAR data testing and HRRP data testing. All these results corroborate that multi-task learning is superior to single tasks learning.

(B) Comparison against the State of the Art

**Figure 13:**
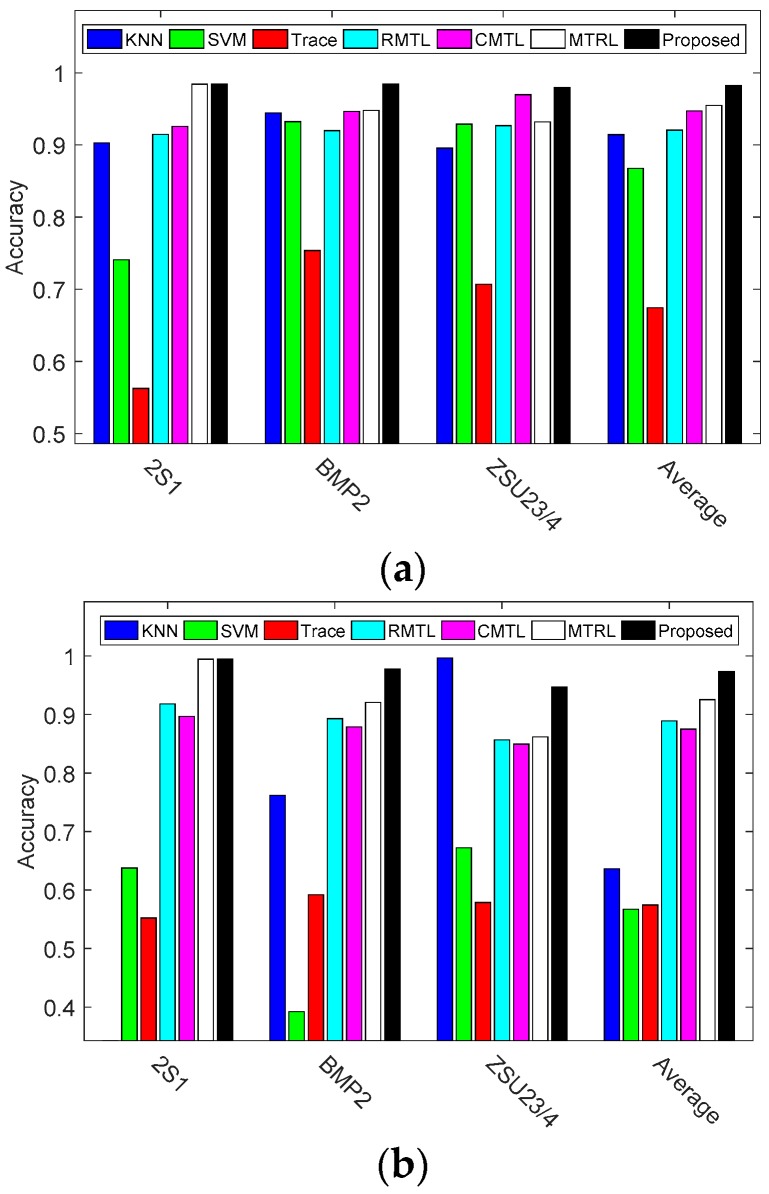
Recognition rates of three vehicles under EOC. (**a**) Testing under 30° depression; (**b**) Testing under 45° depression.

To evaluate the robustness of proposed method, the reference methods are compared with our method under two different depression angles. The ACC results are shown in Figure 13 and Table 7.

The overall recognition rate for the proposed method is 0.9824, compared to 0.9546 for MTRL, 0.9472 for CMTL, 0.9203 for RMTL, 0.6742 for Trace, 0.8673 for SVM and 0.9142 for KNN on the operating condition of 30° depression. It is 2.78%, 3.52%, 6.21%, 30.82%, 11.51% and 6.82% better than MTRL, CMTL, RMTL, Trace, SVM and KNN, respectively. When the algorithms are further evaluated using the images captured at an operating condition of 45°, the performances of all the methods are degraded, especially the KNN method. The recognition rate for KNN drops from 0.9142 to 0.6363. Different form KNN method, the multi-task leaning methods like RMTL, CMTL, MTRL and our method still keep a higher recognition rate due to the ability to share the multi-task relationships. Among all the approaches, the proposed method achieves the highest recognition rate of 0.9731. This is 2.29%, 7.83%, 6.73%, 39.90%, 40.61% and 33.68% better than MTRL, CMTL, RMTL, Trace, SVM and KNN, respectively. The improvement of recognition accuracy is significant. The results demonstrate that the proposed method is much more robust toward depression variation than the reference methods.

From the above extensive analyses of simulation experiment results, we can draw the following conclusions. Multi-task relationships information can actually improve the performance of classification and automatically learning the task relationships from data is a more favorable option. Furthermore, a detailed and accurate description of the multi-task relationships in the original and projected space of model parameters is better than the rough hypothesis of multi-task relationships in terms of improving the radar target recognition performance.

## 4. Conclusions

In this paper, we propose a radar target recognition method based on the theory of clustered multi-task learning. In order to learn more useful and accurate relationships among multiple tasks, the potentially useful relationships in the projection space are further explored. The proposed method assumes that multi-tasks within the same cluster should be close to each other in the original and projected space, which contributes to discriminating the radar targets with similar patterns. Studies on the simulated HRRP data show that the proposed method can fully discover multi-task relationship in the projected space and accurately classify the targets with similar structures. Extensive comparative experiments on the MSTAR data are conducted to further demonstrate the superiority and robustness of the proposed method. The simulation experiment results under SOC and EOC demonstrate that the proposed method can properly reveal the latent relationships among multiple tasks and have a better performance than single task learning. Moreover, all of the comparisons against the state-of-the-art methods indicate the superiority of the proposed method.

## Figures and Tables

**Table 1:** The reference methods to be studied.

Methods	Description
KNN	K-nearest neighbor classifier.
SVM [8]	Support vector machine learning.
Trace-norm Regularized multi-task learning (Trace) [20]	Trace method assumes that all models share a common low dimensional subspace.
Regularized multi-task learning (RMTL) [17]	RMTL method assumes that all tasks are similar, and the parameter vector of each task is similar to the average parameter vector.
Clustered Multi-Task Learning (CMTL) [18]	CMTL assumes that multiple tasks follow a clustered structure and that such a clustered structure is prior. In the experiments, we perform multiple single task learning to get the trained mode parameters, and based on which to obtain the clustered structure.
Multi-task relationship learning (MTRL) [14]	MTRL can autonomously learn the positive and negative task correlation.

**Table 2:** Electromagnetic simulation parameters.

Waveform	Center Frequency	Band-Width	Number of Frequency Samples	Meshing Size	Depression Angles	Azimuth Angles
Chirp Signal	1.5 GHz	1 GHz	1000	λ/6	15°	0–180° with 1° steps

**Table 3:** Recognition rates of all the compared methods.

Method	KNN	SVM	Trace	RMTL	CMTL	MTRL	Proposed
Tank 1	0.8684	0.6929	0.6929	0.9066	0.9822	0.9600	0.9956
Tank 2	0.7237	0.7105	0.6754	0.8844	1.0000	1.0000	1.0000
Tank 3	1.0000	0.8596	0.7149	0.8844	0.8889	0.9522	0.9789
Average	0.8640	0.7543	0.6944	0.8918	0.9570	0.9674	0.9915

**Table 4:** The number of images for training and testing about the ten targets under SOC.

Target	2S1	BRDM2	BTR60	D7	T62	ZIL131	ZSU23/4	BRT70	T72	BMP
Training (17°)	299	298	256	299	299	299	299	233	232(SN_132) 231(SN_812) 228(SN_s7)	233(SN_9563) 232(SN_9566) 233(SN_c21)
Testing (15°)	274	274	195	274	273	274	274	196	196(SN_132) 195(SN_812) 191(SN_s7)	195(SN_9563) 196(SN_9566) 196(SN_c21)

**Table 5:** Recogniton rates of all the compared methods under SOC.

Methods	KNN	SVM	Trace	RMTL	CMTL	MTRL	Proposed
2S1	0.8723	0.8870	0.7082	0.6480	0.7833	0.8860	0.9780
BMP2	0.9590	0.9196	0.7733	0.8571	0.9641	0.9558	0.9665
BRDM2	0.8277	0.9151	0.7082	0.8960	0.9637	0.9757	0.9802
BRT70	0.9541	0.9192	0.7910	0.9674	0.9444	0.9606	0.9674
BTR60	0.9385	0.9113	0.7921	0.9497	0.9016	0.9350	0.9497
D7	0.9781	0.8870	0.7306	0.9806	0.9664	0.9754	0.9806
T62	0.8767	0.8874	0.7316	0.9799	0.9650	0.9731	0.9799
T72	0.9592	0.9192	0.8075	0.9703	0.9670	0.9646	0.9703
ZIL131	0.9197	0.8841	0.7412	0.9806	0.9696	0.9788	0.9806
ZSU23/4	0.9854	0.8870	0.7200	0.9794	0.9658	0.9720	0.9794
Average	0.9271	0.9017	0.7504	0.9209	0.9391	0.9584	0.9734

**Table 6:** The number of images for training and testing about the three targets under EOC.

Target	2S1	BRDM2	ZSU23/4
Training (17°)	299	298	299
Testing (30°)	288	287	288
Testing (45°)	303	303	303

**Table 7:** Recognition rates of all the compared methods under EOC.

Methods	Training (17°)–Testing (30°)				Training (17°)–Testing (45°)			
	2S1	BMP2	ZSU23/4	Average	2S1	BMP2	ZSU23/4	Average
KNN	0.9028	0.9444	0.8955	0.9142	0.1505	0.7617	0.9967	0.6363
SVM	0.7409	0.9322	0.9288	0.8673	0.6373	0.3917	0.6719	0.5670
Trace	0.5625	0.7534	0.7067	0.6742	0.5518	0.5918	0.5787	0.5741
RMTL	0.9144	0.9199	0.9265	0.9203	0.9149	0.9195	0.8830	0.9058
CMTL	0.9254	0.9464	0.9697	0.9472	0.9166	0.8987	0.8692	0.8948
MTRL	0.9841	0.9477	0.9319	0.9546	0.9945	0.9481	0.9082	0.9502
Proposed	0.9841	0.9841	0.9791	0.9824	0.9945	0.9777	0.9472	0.9731
